# Detection of HIV-1 viral load in tears of HIV/AIDS patients

**DOI:** 10.1007/s15010-020-01508-2

**Published:** 2020-08-26

**Authors:** Yujing Qian, Zunyou Wu, Chao Chen, Kuifang Du, Wenbin Wei

**Affiliations:** 1 Beijing Tongren Eye Center, Beijing key Laboratory of Intraocular Tumor Diagnosis and Treatment, Beijing Ophthalmology and Visual Sciences Key Lab, Medical Artificial Intelligence Research and Verification Laboratory of the Ministry of Industry and Information Technology, Beijing Tongren Hospital, Capital Medical University, Beijing, China; 2 National Center for AIDS/STD Control and Prevention, Chinese Center for Disease Control and Prevention, Beijing, China; 3 Beijing You’an Hospital, Capital Medical University, Beijing, China

**Keywords:** Tear, Viral load, HIV, AIDS

## Abstract

**Objectives:**

The tear, as an important bodily secretion, plays a crucial role in preventing infection and maintaining homeostasis of ocular surfaces. Although accumulating studies have reported on the HIV-1 viral load profile among varying bodily fluids and secretions, little was known concerning HIV-1 dynamics in tears. Therefore, the objectives of this study were to investigate the HIV-1 viral load in tears of HIV/AIDS patients and study factors influencing their tear viral load.

**Methods:**

A cross-sectional study was conducted. 67 patients with a confirmed HIV-1 infection or AIDS were recruited from the Beijing You’an Hospital, China between April 2018 and September 2018. Socio-demographic information and laboratory test results were collected. At the same time, ophthalmic examinations were carried out and tear samples were tested.

**Results:**

Of 30 highly active antiretroviral therapy (HAART)-naïve patients, 53.3% had detectable HIV-1 RNA in tears. Of 37 patients on HAART, HIV-1 RNA was undetectable in their tears, regardless of treatment duration and blood viral load. Tear viral load ranged from TND (target not detected) to 13,096 copies/mL. Viral load was lower in tears than in blood plasma (*p* < 0.001), and was significantly correlated with plasma viral load (Rho = 0.566, *p* < 0.001) and AIDS stage (Rho = 0.312, *p* = 0.01), but negatively correlated with CD4 ^+^ T cell count, CD4 ^+^/CD8 ^+^ T cell count, and duration of HIV infection (Rho = -0.450, Rho = − 0.464, Rho = − 0.565; *p* < 0.001).

**Conclusions:**

HIV-1 RNA is present in tears of more than half of the HAART-naïve patients, whereas absent in tears of patients on HAART. Tear viral load is positively associated with plasma viral load while it is negatively correlated with CD4 cell count. This study provides novel insights into the area with limited understanding–HIV-1 viral load in tears.

## Background

It is widely recognized that the HIV-1 virus can penetrate into various tissues and exist in bodily fluids and secretions [1]. Accumulating studies have reported on the HIV-1 viral load profile and associated factors among varying bodily fluids, such as cerebrospinal fluid, saliva, breast milk, and semen [2, 3]. However, such data for tear, an important bodily secretion playing a crucial role in refraction, preventing infection, and maintaining homeostasis of ocular surfaces [4], remains unknown and is worth further investigation.

From the previous since three decades ago, the researcher has found that HIV-1 can be infrequently isolated from tears [5]. Also, it was hard to detect proviral sequences in tears of HIV-positive patients [6]. In 2011, HIV-1 RNA load was reported to be high in tears of patients who underwent long-term highly active antiretroviral therapy (HAART) with undetectable blood viral load, suggesting that the tear-associated tissues could be new reservoirs of HIV-1 [7]. Besides the three above reports, there are few studies regarding the HIV-1 virus in tears, and such available studies were conducted relatively early. Hence, it is essential to investigate the tear HIV-1 viral load in the era of new antiretrovirals. The objectives of this study were to measure the level of HIV-1 RNA in tears of HIV/AIDS patients and study factors influencing their tear viral load.

## Methods

A cross-sectional study was conducted based on 67 eligible patients with a confirmed HIV-1 infection or AIDS diagnosed by infectious disease specialists. These patients were randomly recruited from the Infectious Diseases Center or Ophthalmology Department of the Beijing You’an Hospital, China between April 2018 and September 2018. Among the patients, 30 were HAART-naïve and 37 were HAART-treated, the mean age was 35 ± 9 years. This study was approved by the institutional review board of the National Center for AIDS/STD Control and Prevention, Chinese Center for Disease Control and Prevention. Written consent was obtained according to the Declaration of Helsinki.

Sociodemographic characteristics (e.g. demographic characteristics, mode of HIV-1 transmission, date of HIV-1 diagnosis, medical history, co-infections and opportunistic infections, type of HAART regimen used, and duration of HAART treatment) were collected from all participants. Additionally, participants’ laboratory test results, including plasma HIV-1 viral load, peripheral blood lymphocyte count and the presence of a sexually transmitted infection (STI) were obtained. Ophthalmic examination including best-corrected vision acuity (logMAR) and intraocular pressure were performed. A well-trained ophthalmologist collected at least 0.25 ml (mL) of reflex tears or psychic tears from the conjunctival sac using a micropipette directly into sterile tubes in an ultraviolet-disinfected room [7]. Moreover, quantitative analysis of HIV-1 RNA in tears and blood plasma was performed according to manufacturer’s instructions using Abbott M2000 Real Time HIV-1 assay (Abbott Molecular, Inc.). HIV-1 RNA results were stratified as TND (target not detected), detectable but below 40 copies/mL and linearly quantified above 40 copies/mL.

Data were analyzed using SPSS (IBM Corp. Released in 2017. IBM SPSS Statistics for Macintosh, Version 25.0. Armonk, NY:IBM Corp.). For preliminary data analysis, the Kolmogorov–Smirnov test was applied for normality testing. Variables distributed according to the normal distribution were presented as the mean and standard deviation (SD) and variables that did not apparently follow the normal distribution were reported as the median and interquartile range (IQR). *t* tests were used to compare the means of normally distributed quantitative variables, otherwise, nonparametric Mann–Whitney *U* tests were used to compare the medians of independent non-normal quantitative variables. The nonparametric Wilcoxon signed-rank test was used to compare the medians of two related non-normal variables–tear and plasma viral loads. Chi-square tests were applied to compare qualitative data, such as the detection rate of HIV-1 RNA in tears and blood, and in treatment-naïve patients and HAART-treated patients. Spearman’s rho test was computed for measuring associations of tear viral load. *p* < 0.05 was considered statistically significant.

## Results

Of 67 total participants, the mean CD4^+^ T cell count was 466 cells/μL [standard deviation (SD) 276 cells/μL] and the median CD4 ^+^/CD8 ^+^ T cell count was 0.48 (range 0–1.87) (Table 1). Treatment-naïve patients had a lower CD4 ^+^ T cell count and CD4 ^+^/CD8 ^+^ T cell count compared to HAART-treated patients (315 vs. 589 cells/μL, 0.29 vs. 0.57, *p* < 0.001, respectively). Of 37 patients who were on HAART, the median duration of infection and treatment were 36 months (range 1–191) and 34 months (range 1–146). 22 of 37 (59.5%) patients on TDF (tenofovir disoproxil fumarate) + 3TC (lamividine) + EFV (efavirenz), 5/37 (13.5%) on TDF + 3TC + DTG (dolutegravir) and 5/37 (13.5%) on TDF + 3TC + LPV/r (lopinavir/ritonavir), respectively.

**Table 1:** Characteristics and viral load detection levels by the total participant population and treatment status

	Total (*n* = 67)	Treatment-naïve patients (*n* = 30)	HAART-treated patients (*n* = 37)	*p*-value
Age (years), $\overline{x} \pm s$	35 ± 9	36 ± 10	35 ± 9	0.853^b^
Male, *n* (%)	65 (97.0)	30 (100.0)	35 (94.6)	0.498^c^
Homosexual, *n* (%)	49 (73.10)	22 (73.33)	27 (73.00)	0.971^c^
Co-infection, *n* (%)	42 (62.69)	22 (73.33)	20 (54.05)	0.105^c^
AIDS stage, *n* (%)*	14 (20.90)	11 (36.67)	3 (8.11)	0.004^c^
LogMAR vision acuity, M (IQR)	0.00 (0.00–0.10)	0.00 (0.00–0.06)	0.00 (0.00–0.10)	0.797^a^
IOP (mmHg), M (IQR)	13.50 (12.50–15.50)	13.25 (12.00–15.13)	14.50 (13.25–15.75)	0.037^a^
Duration of HIV-1 infection* (months), M (IQR)	10.00 (0.00–56.00)	0.00 (0.00–4.50)	36.00 (10.00–85.00)	< 0.001^a^
CD4^+^T cell count* (cells/μL), $\overline{x} \pm s$	466 ± 276	315 ± 229	589 ± 252	< 0.001^b^
CD4/CD8*, M (IQR)	0.48 (0.24–0.62)	0.29 (0.15–0.49)	0.57 (0.44–0.80)	< 0.001^a^
Blood viral load* (copies/mL), M (IQR)	156 (0–21,539)	22,555(4350.5–52,593)	0 (0–40)	< 0.001^a^
Tear viral load (copies/mL), M (IQR)	0 (0–0)	40 (0–447.5)	0 (0–0)	< 0.001^a^
Detection rate of blood HIV-1 RNA (%)	62.7	100.0	32.4	< 0.001^c^
Detection rate of tear HIV-1 RNA (%)	23.9	53.3	0.0	< 0.001^c^
Duration of HAART treatment (months), $\overline{x} \pm s$			43 ± 36	

*HIV* human immunodeficiency virus, *RNA* ribo nucleic acid, *M (IQR)* Median interquartile range, *LogMAR* logarithm of the minimum angle of resolution, *IOP* intraocular pressure, *mmHG* millimeters of Mercury, *HAART* highly active antiretroviral therapy, *mL* milliliters

*Factors associated with tear viral load in tears among all participants

^a^Mann–Whitney *U* test

^b^Independent samples *t* test

^c^Chi-square test

Of the 67 subjects, 62.7% of patients had detectable HIV-1 RNA in blood plasma compared to 23.9% who had detectable HIV-1 RNA in tears (*p* < 0.001). Of 30 HAART-naïve patients, 100.0% had detectable HIV-1 viral load in blood in contrast to 53.3% of whom had detectable HIV-1 RNA in their tears (*p* < 0.001). Strikingly, of those on HAART therapy, 32.4% of patients had detectable HIV-1 RNA in blood, while HIV-1 RNA was detectable in tears in 0.0% of treated participants (*p* < 0.001). Additionally, of all participants, the maximum tear viral load reached to 13,096 copies/mL. Median viral load was lower in tears than in blood plasma in all patients, HAART-naïve patients and HAART-treated patients, respectively (0 vs. 156 copies/mL, 40 vs. 22,555.5 copies/mL, 0 vs. 0, *p* < 0.001).

Additionally, 14 of 67 participants (20.9%) were in AIDS stage. Amongst, 11 AIDS patients were HAART-naïve. The proportion of AIDS patients was significantly higher in HAART-naïve group than in HAART-treated group (36.7% vs. 8.1%, *p* = 0.004). Furthermore, compared to HIV-1-infected patients, AIDS patients had a higher level of blood viral load (40 vs. 21,366.5 copies/mL, *p* < 0.001) and tear viral load (0 vs. 20 copies/mL, *p* = 0.011), but a much lower CD4 ^+^ T cell count and CD4 ^+^/CD8 ^+^ T cell count (542 vs. 75 cells/μL, 0.51 vs. 0.13, *p* < 0.001).

Moreover, tear viral load was found to be associated with blood viral load (Rho = 0.566, *p* < 0.001), CD4^+^ T cell count (Rho = − 0.450, *p* < 0.001), CD4^+^/CD8^+^ T cell count (Rho = − 0.464, *p* < 0.001), duration of HIV-1 infection (Rho = − 0.565, *p* < 0.001), AIDS stage (Rho = 0.312, *p* = 0.01), and intraocular pressure (Rho = − 0.252, *p* = 0.039). No other variables (e.g. volume of tear samples, dilution multiple, age, CD8^+^ T cell count, co-infection) were significantly associated with tear HIV-1 RNA level (data not shown).

## Discussion

With a better understanding of HIV persistence in several bodily fluids, it is essential to explore the HIV-1 viral load profile in a greater variety of bodily fluids, such as tears, as it is especially critical for ophthalmologists. However, few studies have focused on this issue. Our study aimed to gather baseline information regarding the presence of HIV-1 RNA viral load in tears and its associated factors. The results of this study can also be used to discuss the effect of current antiretroviral regimens in eliminating tear HIV-1 virus and the existence of potential HIV-1 reservoirs in eyes.

Among published articles, we haven’t found data documenting the detection rate of HIV-1 RNA in tears of treatment-naïve patients. In addition, little existing research was known to investigate the factors that affect tear viral load. Our study reported the presence of tear HIV-1 RNA in 53.3% of treatment-naïve patients. Moreover, the detection rate and viral load were lower in tears than in blood plasma. In addition, the present study demonstrated that tear viral load was associated with blood viral load, CD4^+^ T cell count, CD4^+^/CD8^+^ T cell count, duration of HIV-1 infection. Tear viral load was higher in AIDS patients than in HIV-1-infected patients. Although these findings are well-known among several bodily fluids and secretions [8, 9], it is new in tears, adding innovative value on an area with limited understanding– HIV-1 viral load in tears. The current results also address the necessity for ophthalmologists to take precautions while performing ocular examinations and surgeries in HIV/AIDS patients.

Interestingly, we also found HIV-1 RNA was undetectable in tears of all participants who were on HAART therapy (treatment duration ranging from 1 to 146 months). Additionally, the detection rate and viral load in tears of HAART-treated patients were significantly lower than untreated patients. These findings may provide us two valuable information. First, we notice that our results are opposing the study by Han et al. [7]. Since the previous study did not show its detailed treatment regimens, from our present data, we speculate that the neutralization of tear HIV-1 virus primarily depends on the current strategies of early treatment initiation and potent HAART therapy [10]. Second, the eyes are mostly considered as an immune-privileged organ that may inhibit the effect of antiretroviral regimen, nevertheless, our results indicate that current antiretroviral drugs could penetrate through the blood-ocular barrier and suppress HIV-1 in tear-associated tissues effectively.

Furthermore, while a great number of studies have discovered the discordant relationship between HIV-1 viral load in blood and several bodily fluids, suggesting the existence of viral reservoirs such as the lacrimal gland, genital tract, and central nervous system [7, 11, 12], however, in this study, 25/37 patients who were on HAART had undetectable HIV-1 RNA in their blood plasma, together with undetectable tear viral load consistently. Consequently, our data question the view that tear-associated tissues could represent as new viral reservoirs.

The current study was somewhat limited by the cross-sectional nature, thus we were unable to assess the causality. Besides, the small sample size and the small volume of tear samples for polymerase chain reaction analysis are also limitations of this study, even though these two parameters did not influence the test results according to the statistical analysis. Further longitudinal studies, which compare the HIV-1 RNA viral load, HIV-1 RNA sequences, and anti-retroviral drug concentration in tears over time, could help inform the mechanism of HIV infection in eyes.

In summary, HIV-1 RNA is present in tears of more than half of the HAART-naïve patients, whereas absent in tears of patients on HAART. Tear viral load is positively associated with plasma viral load while it is negatively correlated with CD4 cell count. Our study provides further evidence for the effectiveness of current antiretroviral regimens in eyes. Nevertheless, we question about the idea of considering the lacrimal gland as a new HIV reservoir. This study adds novel understanding about HIV-1 viral dynamics in tears and provides a new perspective that deserves more attention in future investigations.
